# Supplementary material for: Journal editors’ perspectives on the roles and tasks of peer reviewers in biomedical journals: a qualitative study
Source: BMJ Open. 2019 Nov 24;9(11):e033421. doi: 10.1136/bmjopen-2019-033421 (PMC6886905; doi:10.1136/bmjopen-2019-033421)
Supplement: Supplementary data [file bmjopen-2019-033421supp001.pdf]

Topic guide for semi-structured interviews

Key area of investigation	Topics	Questions and prompts
Background information	- Explore personal background<br>- Level of experience<br>- Roles and tasks as an editor	• Tell me about your journal and the job you have.<br>• How long have you been in this position?<br>• Did you hold any other editorial position before your current position? If yes, what were your responsibilities then?<br>Prompt: percentage of time devoted to editorial duties (e.g., part time, full time)<br>• What are your current responsibilities (roles and tasks)?
Journal set-up	- Explore journal set-up	• Tell me about your journal - how does it work?<br>Prompt: availability of resources (e.g. human and financial resources), relationship with publisher<br>• How does the peer review process work in your journal?<br>Prompt: submission system, peer review model (e.g., single blind etc)<br>• What do you do within the process?<br>Prompt: Interaction with peer reviewers
Opinion on peer reviewers roles and tasks	- Roles and tasks of peer reviewers<br>- Expectations	• What do you expect from peer reviewers in terms of their roles and tasks?<br>• What about training for peer reviewers?<br>Prompt: use items from scoping review (roles and task related), attitudes and beliefs (e.g. on training, how they peer review themselves) , organisational expectations<br>• How do you let your reviewers know what you expect from them?<br>Prompt: journal guidelines<br>• Can you tell me about a specific situation when you were not satisfied with a review report or with a peer reviewer?<br>• What did you do in that a situation?<br>Prompt: probe for factors other than being late with a review, or not doing a review once you they have accepted it<br>• Can you tell me about a situation when you were exceptionally satisfied with a review or with a peer reviewer?<br>• Were there situations (in regards to the roles and task of reviewers) when you disagreed with the other editors you work with? What about? What happened?<br>• What about other journals, do roles and tasks differ among journals in your field?<br>Prompt: if yes (i.e. differences exist), then:<br>• How does this affect the process?<br>• How does it affect your communication?<br>• How do you negotiate those differences? Does it matter?

Communication between editors, peer reviewers and authors	- Communication between the three parties<br>- Potential conflicts<br>- Power	• Can you describe your experience of the communication process between editors, authors and peer reviews?<br>• How do you communicate with authors and peer reviewers?<br>• Can you give me some specific examples of situations where this communication is challenging?<br><br>Prompt:<br>What are potential conflicts?<br>When do disagreements arise?<br>What happens if there is disagreement between peer reviewers?
Conclusion	- Snowballing<br>- Documents<br>- Final comments	• Is there anybody else whom you think I should speak to?<br>• Any articles/documents I can access/should look at?<br>• Any final comments? Is there anything else that you think is important to mention?
