# Supplementary material for: Journal editors’ perspectives on the roles and tasks of peer reviewers in biomedical journals: a qualitative study
Source: BMJ Open. 2019 Nov 24;9(11):e033421. doi: 10.1136/bmjopen-2019-033421 (PMC6886905; doi:10.1136/bmjopen-2019-033421)
Supplement: Supplementary data [file bmjopen-2019-033421supp002.pdf]

Actions undertaken to establish trustworthiness of analysis

Phases of thematic analysis	Techniques for establishing trustworthiness
Phase 1:<br>Familiarizing with the data	**Prolonged engagement with data** (credibility)<br>- KG performed multiple readings of all transcripts<br>**Reflexive journaling** (confirmability)<br>- Documentation of thoughts and potential codes/themes were taken during phase 1 and throughout the entire data analysis
Phase 2:<br>Generating initial codes	**Member-checking and peer debriefing** (credibility)<br>- The first six interviews were coded independently by two researchers (KG and DH) leading to the creation of the initial codebook<br>**Audit trail** (confirmability)<br>- Codebook was updated after every new interview creating an audit trial of the code generation
Phase 3:<br>Searching for themes	**Researcher triangulation** (confirmability)<br>- Regular team meetings to review findings from different perspectives<br>- Diagramming/drawing to make sense of theme connections
Phase 4:<br>Reviewing themes	**Researcher triangulation** (confirmability)<br>- Regular team meetings to vet themes and subthemes<br>**Audit trail** (confirmability)<br>- We returned to raw data to check for referential adequacy
Phase 5:<br>Defining and naming themes	**Researcher triangulation** (confirmability)<br>- Team consensus on themes
Phase 6:<br>Producing the report	**Thick description** (transferability)<br>- The methodological approach and analytical choices were described in detail in previously published study protocol<br>- We provide detailed descriptions of study results<br>- Peer debriefing with researchers outside of the core group (IB and DM)
