# Supplementary material for: Journal editors’ perspectives on the roles and tasks of peer reviewers in biomedical journals: a qualitative study
Source: BMJ Open. 2019 Nov 24;9(11):e033421. doi: 10.1136/bmjopen-2019-033421 (PMC6886905; doi:10.1136/bmjopen-2019-033421)
Supplement: Supplementary data [file bmjopen-2019-033421supp003.pdf]

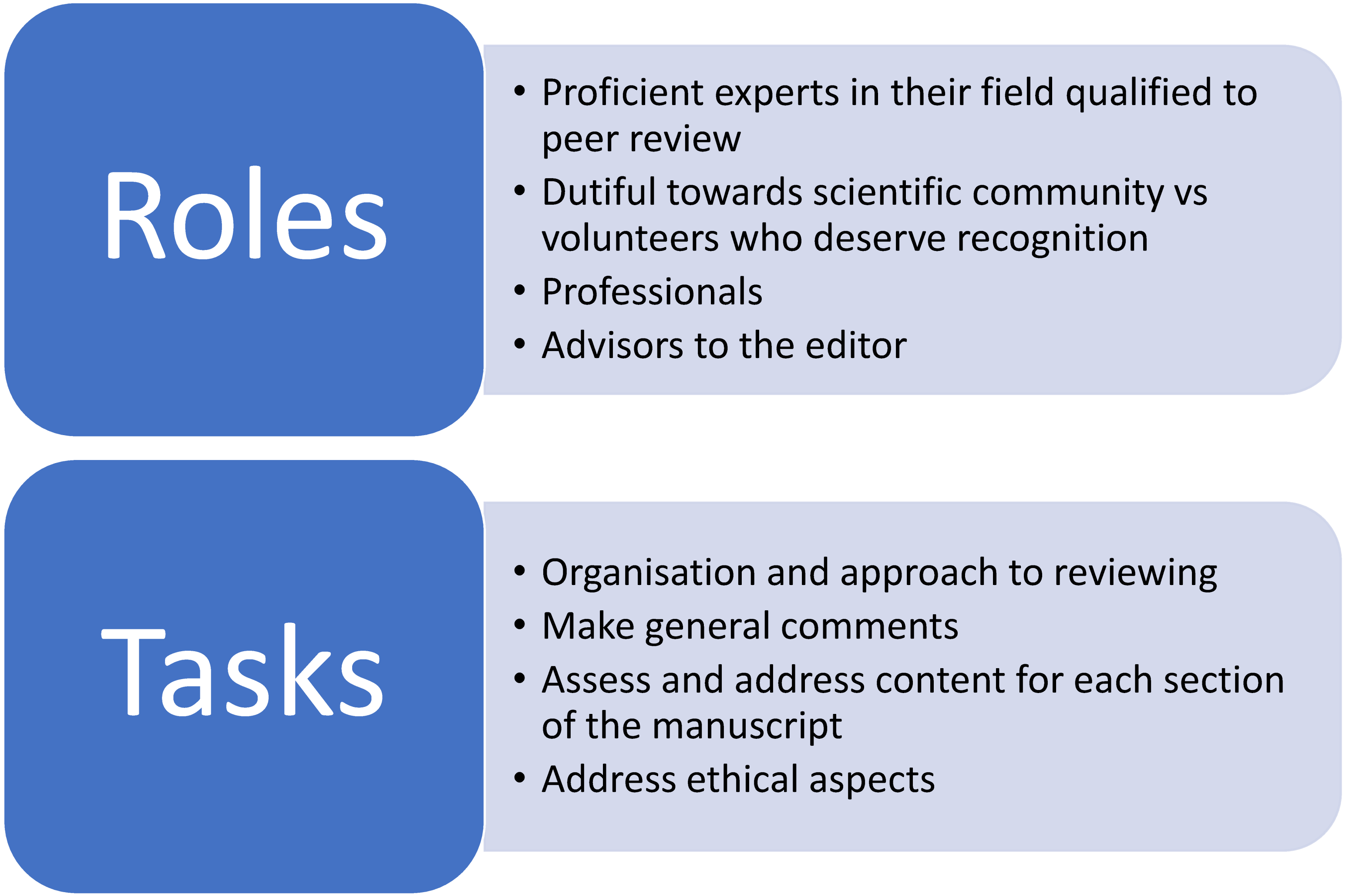

Additional file 3

Roles and Tasks of peer reviewers
